# Neurological Responses to Scented Insects via Olfactory Stimulation: A Controlled fMRI Study

**DOI:** 10.3390/insects17080761

**Published:** 2026-07-24

**Authors:** Hee-Eun Hong, Hansol Lee, Hae-Jin Ko, Ji-Yeon Park, A-Sol Kim, Ji-Eun Song, Yongmin Chang, Sangmin Ji, Kwanho Park

**Affiliations:** 1 Department of Family Medicine, Kyungpook National University Hospital, Daegu 41944, Republic of Korea; honghin@knuh.kr (H.-E.H.); miniev@naver.com (J.-Y.P.); 2 Department of Medical & Biological Engineering, Kyungpook National University, Daegu 41566, Republic of Korea; leehs9836@naver.com; 3 Department of Family Medicine, School of Medicine, Kyungpook National University, Daegu 41405, Republic of Korea; deepai@knu.ac.kr (A.-S.K.); love2uje@naver.com (J.-E.S.); 4 Department of Family Medicine, Kyungpook National University Chilgok Hospital, Daegu 41404, Republic of Korea; 5 Department of Molecular Medicine, School of Medicine, Kyungpook National University, Daegu 41405, Republic of Korea; ychang@knu.ac.kr; 6 Department of Radiology, Kyungpook National University Hospital, Daegu 41944, Republic of Korea; 7 Industrial Entomology Division, Department of Agricultural Biology, National Institute of Agricultural Sciences, Jeonju 55365, Republic of Korea; jeee3ang@korea.kr (S.J.); nicegano@korea.kr (K.P.)

**Keywords:** animal-assisted therapy, brain, emotions, insects, magnetic resonance imaging, memory, neurologic response, olfactory perception

## Abstract

The link between the olfactory system and psychiatric disorders is well documented; however, the neurological effects of insect-derived scents on human brain function remain unexplored. This study examined the neurological response to scented insects using functional magnetic resonance imaging (fMRI) to evaluate their potential as a novel animal-assisted intervention (AAI) for mental health. In this controlled, crossover trial, the olfactory effects of scented insects (*Poecilocoris splendidulus* Esaki) were compared with a control scent (alcohol) in 29 psychologically healthy adults. Brain activation differences were assessed using paired *t*-tests with false discovery rate correction, followed by multiple regression analyses incorporating psychometric assessments. Compared with the control scent, the insect scent elicited greater activation across sensory, emotional, and memory-related brain networks, including the thalamus, insula, hippocampus, and prefrontal cortex (*p* < 0.05). Furthermore, individual psychometric variations influenced these responses: higher depressive symptoms were associated with reduced activation in the anterior cingulate cortex, while elevated stress and anxiety correlated with heightened activity in the hippocampus and inferior frontal gyrus. These findings suggest that insect-derived scents induce distinct neurological responses linked to emotional processing, supporting their potential application in therapeutic interventions for mental health.

## Simple Summary

The connection between our sense of smell and mental health is well established, but how insect-derived scents affect the human brain remains unknown. This study used functional magnetic resonance imaging to examine brain responses to a natural insect scent in twenty-nine healthy adults. Compared to an alcohol-based control scent, the insect scent significantly increased activity in brain regions responsible for sensory integration, emotion regulation, and memory. Furthermore, these brain responses varied depending on individual psychological traits, such as levels of depression, anxiety, and stress. These findings suggest that insect-derived scents can influence emotional and cognitive brain networks. This research provides an important foundation for future non-contact, insect-based approaches aimed at promoting mental health and psychological well-being.

## 1. Introduction

Animal-assisted interventions (AAIs), defined as “any intervention that intentionally includes or incorporates animals as part of a therapeutic or ameliorative process or milieu,” have demonstrated psychological and physical benefits across diverse populations [1]. In terms of psychological health, AAI has demonstrated a high potential in the treatment of psychiatric disorders among young and adult patients [2,3,4]. As a subtype of AAI, pet insects have also been demonstrated to exert beneficial effects on the psychological health of elderly individuals. Studies indicate positive effects of these insects on depression and cognitive function in community-dwelling elderly people, as well as improvements in physical function and sleep in prefrail and frail elderly adults [5,6]. Moreover, a functional magnetic resonance imaging (fMRI) study found that rearing pet insects increased activation in the right dorsolateral prefrontal cortex and parietal cortex of elderly women with a low baseline executive function, suggesting enhanced cognitive performance [7].

The olfactory system is closely linked to neurodegenerative and psychiatric disorders, and specific scents can modulate mood, cognition, and emotion [8,9,10]. Aromatherapy, involving the inhalation of aromatic plant extracts or oils, has shown therapeutic effects on pain, insomnia, anxiety, stress and even cognitive function [11,12,13,14]. Additionally, specific scents such as jasmine or natural odors have been shown to affect mood [15,16]. However, prior studies have focused primarily on plant-derived scents, with only limited exploration of animal-derived olfactory stimuli.

Compared with traditional companion animals, insects offer several practical advantages as potential non-contact animal-assisted interventions, including low maintenance burden, minimal safety concerns, and greater accessibility for elderly populations and urban environments. Furthermore, unlike conventional aromatherapy, which primarily relies on pleasant plant-derived scents, insect-derived odors may represent a relatively unfamiliar olfactory stimulus capable of evoking distinct patterns of emotional and cognitive neural processing. Despite these potential advantages, no research has explored the potential effects of insect-derived scents on humans.

Brain regions activated in response to scent stimulation vary depending on the type and quality of the scent. Olfactory information carried by different scents elicits localized brain activation not only in primary olfactory regions but also in areas involved in emotion, memory, and cognition. fMRI is a widely used method for delivering olfactory stimuli and identifying the corresponding brain responses. These responses involve changes in local cerebral blood flow, resulting in two types of MRI signals: perfusion MRI signals, which directly reflect cerebral blood flow, and blood oxygenation level–dependent (BOLD) changes, which reflect secondary hemodynamic responses. Among these, fMRI with BOLD contrast has been particularly valuable for mapping brain functions, including those elicited by olfactory scent–based stimuli [17,18].

Given the lack of studies on the neurological effects of insect-derived scent stimulation in humans, this study represents, to our knowledge, the first controlled, crossover fMRI investigation exploring brain responses to scented insects. The primary aim of this study was to identify the specific neural regions involved in processing insect-derived scents. The secondary aim was to determine whether insect-derived scents modulate mood, cognition, and emotional states by examining the relationship between neural activation in these regions and the psychometric scores of the participants. By elucidating how these scents influence brain function, we sought to establish a foundation for future research on the potential of scented insects as a novel AAI for improving mental health.

## 2. Methods

### 2.1. Study Design and Participants

This controlled, crossover trial investigated neural responses to scented insects versus a control scent. Recruitment of the participants began in May 2024, and their MRI scans were completed between July and August 2024. Participants were healthy adults aged 20–49 years without MRI contraindications. Exclusion criteria included: (i) individuals with contraindications to MRI, such as those with a ferromagnetic pacemaker, defibrillator, cochlear implant, insulin pump, or cerebral aneurysm clip; (ii) individuals with metallic implants or prostheses that could interfere with imaging (e.g., post-implant surgery, artificial joints); (iii) individuals currently taking psychiatric medications, including benzodiazepines, methylphenidate, antipsychotics, or Z-drugs; (iv) individuals diagnosed with or undergoing treatment for major psychiatric or neurological conditions, such as major depressive disorder, bipolar disorder, anxiety disorders, schizophrenia, or uncontrolled epilepsy; (v) individuals with entomophobia; (vi) individuals with claustrophobia; (vii) individuals with significant olfactory dysfunction; and (viii) individuals who withdrew consent during the study.

The study protocol was reviewed and approved by the Institutional Review Board of Kyungpook National University Hospital (IRB No. KNUH 2024-05-017).

During the study period, 37 participants were recruited after screening for exclusion criteria through direct interviews. All 37 completed psychometric assessments and the first fMRI session. Thereafter, seven participants were excluded for exceeding the predefined cutoff scores for depression and anxiety, and one participant was excluded due to unsuccessful fMRI data acquisition caused by dental braces. Consequently, 29 psychologically healthy adults, with psychometric scores within normal ranges, successfully completed both fMRI sessions.

### 2.2. Demographic Data and Psychometric Assessment

Basic demographic information, including age, underlying medical conditions, and current medications, was collected for all participants. Eligibility was confirmed based on the predefined exclusion criteria. Participants then underwent a structured interview and completed standardized psychometric assessments for the evaluation of depression, stress, anxiety, and insomnia.

Depression was assessed using the Patient Health Questionnaire-9 (PHQ-9), with scores of 0–4, 5–9, 10–19, and ≥20 indicating normal, mild, moderate, and severe depression, respectively. In this study, a score of ≥10 was used as the threshold for clinically relevant depression [19].

Stress was evaluated using the Brief Encounter Psychosocial Instrument (BEPSI), which assesses interactional models of stress. The instrument comprises five items rated on a 5-point Likert scale. Average scores were interpreted as follows: <1.8, low stress; 1.8–2.8, moderate stress; and >2.8, high stress. In this study, a score of ≥2.4 was considered indicative of high stress [20,21].

Anxiety was measured using the Beck Anxiety Inventory (BAI). Scores of 0–7, 8–15, 16–25, and 26–63 indicated normal, mild, moderate, and severe anxiety, respectively. In this study, a score of ≥16 was considered indicative of clinically significant anxiety [22,23].

Insomnia was assessed using the Insomnia Severity Index (ISI), a 7-item screening tool. Scores of 0–7, 8–14, 15–21, and 22–28 indicated no insomnia, subthreshold, moderate, and severe insomnia, respectively. A score of ≥15 was considered indicative of clinically significant insomnia [24].

Participants also reported average nightly sleep duration over the past 2 weeks in response to the open-ended question: “How many hours did you sleep on an average night during the recent 2 weeks?” Self-reported sleep duration correlates well with objective measures such as actigraphy and polysomnography [25]. Because depression and anxiety can influence olfactory identification and detection [26,27,28], participants were screened for these conditions using the PHQ-9 and BAI, respectively. Participants with PHQ-9 scores of ≥10 (moderate depression) and BAI scores of ≥16 (moderate anxiety) were excluded from the analysis. These thresholds were derived from validated cutoff values widely adopted in psychiatric research [19,22,23].

### 2.3. Scented Insects

Following the recommendation of entomologists, the great clown stink bug (*Poecilocoris splendidulus* Esaki) was selected as the scented insect for this study. Native to the central and southern regions of Korea, this species emits a distinct but faint natural scent. It measures 17–20 mm in length and is recognized by red markings on a pale green background [29]. The insect typically inhabits boxwood and arborvitae trees and is rarely encountered in urban areas, with contact generally limited to insect farmers or specialists. Prior to this study, none of the participants had previously seen or been exposed to the scent of this insect.

### 2.4. Olfactory Stimulation Method

To compare neural effects, alcohol was used as the control scent. A custom cylindrical inhaler (~30 × 80 mm, 30 mL capacity) was designed for comfortable handling, with a top-positioned filter and an opaque exterior to eliminate visual cues.

Either scented insects or cotton balls soaked in alcohol were inserted inside the inhaler, with a layer of loosely structured sponge on top to diffuse the scent. During fMRI scanning, participants lay in a supine position and, upon verbal instruction, held the inhaler close to one nostril to inhale the scent (additional procedural details are provided below). For the scented insect condition, approximately 10 live *Poecilocoris splendidulus* were placed inside the container immediately before scanning, as the insects emit a distinct natural scent while alive. For the control condition, three to five cotton balls soaked in alcohol were inserted into the inhaler to match the intensity of the insect’s scent.

After completing each scent exposure during the fMRI sessions, participants rated their preference for the scent using a 5-point Likert scale (1 = strongly dislike, 2 = dislike, 3 = neutral, 4 = like, and 5 = strongly like).

### 2.5. MRI Data Acquisition

**Figure 1:**
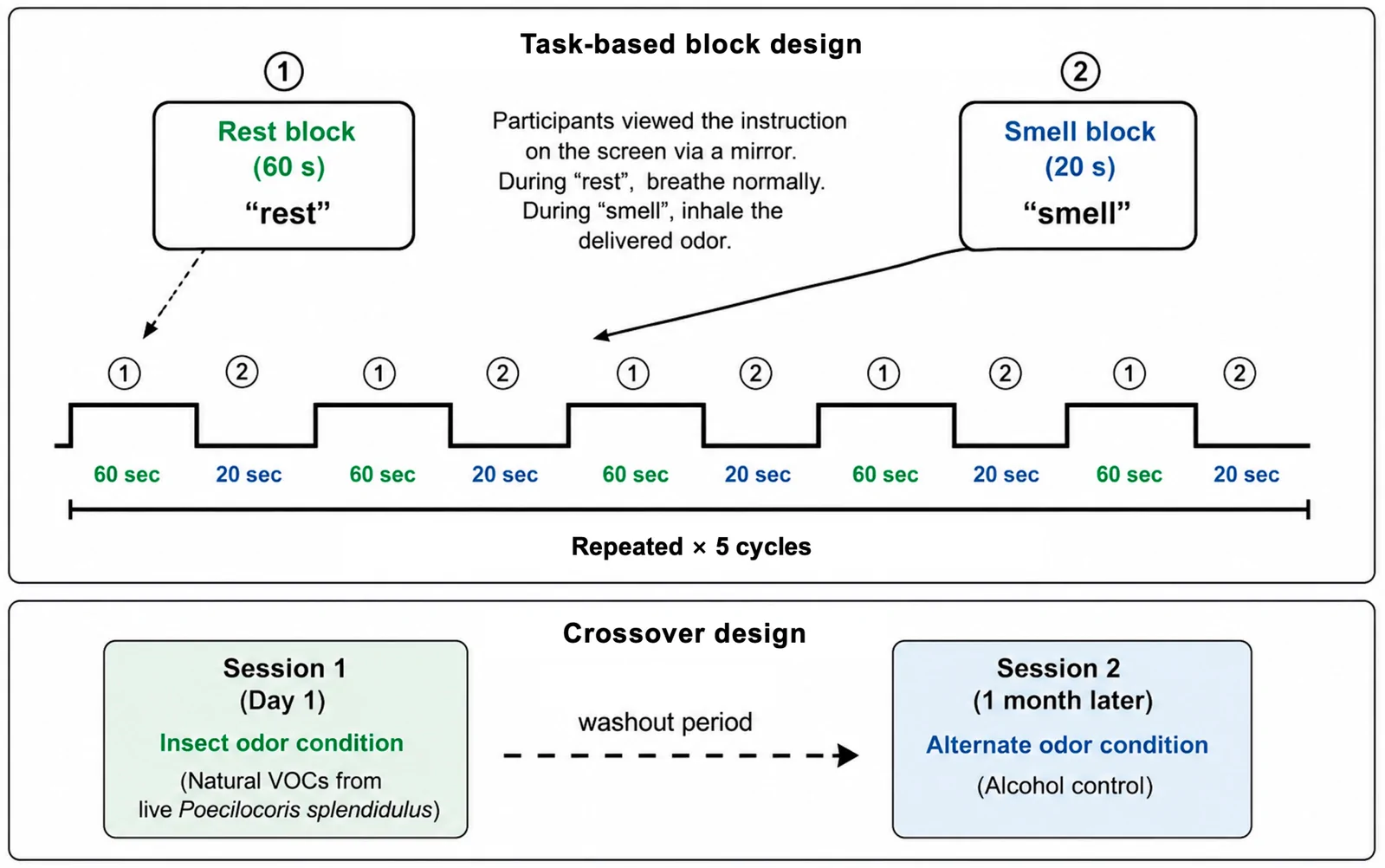
fMRI imaging paradigm. fMRI data were acquired using a block-design task consisting of a 60-s rest period followed by 20-s of scent exposure (scented insect or control scent). This rest–scent cycle was repeated five times per session. The alternate scent condition was applied 1 month later.

All participants underwent two olfactory fMRI sessions under a task-based block-design paradigm. To ensure rigorous sensory isolation, we custom-designed an opaque cylindrical inhaler connected to a clinical-grade inhalation mask, ensuring that participants were exposed only to the olfactory stimulus without any visual cues. During each MRI session, participants lay in a supine position in the scanner with their heads secured, while a mirror mounted on the head coil allowed them to view task instructions projected onto an in-bore screen. First, a 2-min localization process was performed, followed by a 5-min T1-weighted structural scan. Immediately thereafter, the 7-min functional task run commenced, which was precisely synchronized with the visual prompts on the screen. Specifically, during the 60-s Rest Block, participants viewed a “rest” prompt and remained still with the mask on, breathing normally without any odor delivery. This was followed by the 20-s Smell Block, during which the appearance of the “smell” instruction prompted participants to inhale the delivered scent, consisting of either the natural volatile organic compounds from live *Poecilocoris splendidulus* or the alcohol control. This 80-s cycle, comprising a 60-s rest followed by a 20-s smell, was repeated continuously five times per session. To prevent carryover effects and sensory adaptation, the crossover session with the alternate scent was administered on a separate day, one month later (Figure 1). All imaging data were acquired using a 3.0 Tesla MRI scanner (Signa Architect, GE HealthCare, Chicago, IL, USA) equipped with a 48-channel head coil. Functional images were obtained through T2*-weighted echo planar imaging (EPI) with the following parameters: repetition time (TR) = 2000 ms, echo time (TE) = 30 ms, flip angle (FA) = 90°, field of view (FOV) = 24 cm, and acquisition matrix = 64 × 64. Anatomical brain images were acquired using T1-weighted brain volume imaging (BRAVO) with the following parameters: TR = 7.7 ms, TE = 3.1 ms, FA = 12°, FOV = 25.6 cm, and acquisition matrix = 256 × 256.

### 2.6. MRI Data Analysis and Statistical Analysis

#### 2.6.1. Preprocessing Pipeline

Functional images were preprocessed using SPM12 (revision 7771, Wellcome Centre for Human Neuroimaging, London, UK; https://www.fil.ion.ucl.ac.uk/spm/software/spm12/, accessed on 21 July 2026) and CAT12.9 (revision 2560, Structural Brain Mapping Group, Jena, Germany; https://neuro-jena.github.io/cat/, accessed on 21 July 2026) according to the following sequential steps:

1. Slice Timing Correction: Corrected for temporal differences in slice acquisition.
2. Realignment: Corrected for head motion by rigid-body transformation. Participants with head displacement > 2.0 mm or rotation > 2.0° were excluded.
3. Co-registration: The realigned functional images were co-registered to each participant’s high-resolution T1-weighted structural image.
4. Segmentation and Normalization: Structural T1 images were segmented into gray matter, white matter, and cerebrospinal fluid using CAT12. The estimated parameters were subsequently applied to normalize the functional images into the Montreal Neurological Institute (MNI) standard space.
5. Spatial Smoothing: Normalized images were smoothed using an 8-mm full-width at half-maximum (FWHM) Gaussian kernel to improve the signal-to-noise ratio.

#### 2.6.2. First-Level and Second-Level Statistical Analysis

- First-level (Individual) Analysis: A General Linear Model (GLM) was applied to each participant. The design matrix modeled the smell blocks (20 s) and rest blocks (60 s) using a box-car function convolved with the canonical hemodynamic response function (HRF). Six head-motion parameters were included as nuisance regressors to minimize residual motion-related effects. Individual contrast maps (Scented Insect > Rest and Control Scent > Rest) were generated for each participant.
- Second-level One-sample *t*-test: Individual contrast maps were entered into voxel-wise one-sample *t*-tests separately for the scented insect and control scent conditions to identify brain regions significantly activated within each condition.
- Second-level Paired *t*-test: To identify brain regions showing greater activation during exposure to the scented insect odor than during the control scent, individual contrast maps from the two conditions were entered into a voxel-wise paired *t*-test (Scented Insect > Control Scent). Statistical significance was determined using a false discovery rate (FDR)-corrected threshold of *p* < 0.05.
- Multiple Regression Analysis: Multiple regression analyses were subsequently performed using PHQ-9, BEPSI, BAI, and ISI scores as covariates. Each psychometric score was entered separately into an individual regression model. The resulting statistical maps were masked using the paired *t*-test activation map (FDR-corrected, *p* < 0.05), and only voxels overlapping with the paired *t*-test results were retained. Statistical significance within the masked regions was assessed at an uncorrected threshold of *p* < 0.05. Regions of interest (ROIs) were defined as 4-mm-radius spheres centered at the peak *t*-value of each significant cluster from the masked multiple regression results. Beta values extracted from these ROIs were subsequently used for correlation analyses with the psychometric assessment scores.

## 3. Results

### 3.1. Participant Characteristics and Scent Preference Ratings

Among the 29 participants included in the final analysis, 72.4% (*n* = 21) were female, and 27.6% (*n* = 8) were male, with a mean age of 28.2 years. The overall mean psychometric scores were as follows: PHQ-9, 2.8; BEPSI, 1.6; BAI, 3.9; and ISI, 4.5. The average daily sleep duration was 7.2 h. All values were within established normative ranges.

Scent preferences were rated immediately after each fMRI session. The results from the total sample (*n* = 29) showed that the average preference rating was 4.0 (“like”) for the scented insect stimulus and 2.8 (“neutral”) for the control scent (*p* < 0.001; Table 1).

### 3.2. Brain Activation in Response to Scented Insect Versus Control Scent

To characterize the neural responses elicited by each scent condition, voxel-wise one-sample *t*-tests were first performed separately for the scented insect and control scent conditions. The corresponding activation maps and peak coordinates are presented in Supplementary Figures S1 and S2 and Supplementary Tables S1 and S2, respectively.

The one-sample analyses demonstrated that exposure to the scented insect elicited activation across multiple cortical and subcortical regions, including the amygdala, anterior and middle cingulate cortices, caudate, hippocampus, parahippocampal gyrus, insula, inferior and middle frontal gyri, Rolandic operculum, precuneus, and parietal regions (Supplementary Figure S1 and Supplementary Table S1). In contrast, the control scent elicited a relatively limited activation pattern that was primarily confined to the left supramarginal gyrus, inferior and superior parietal lobules, and the right middle cingulate cortex (Supplementary Figure S2 and Supplementary Table S2).

**Figure 2:**
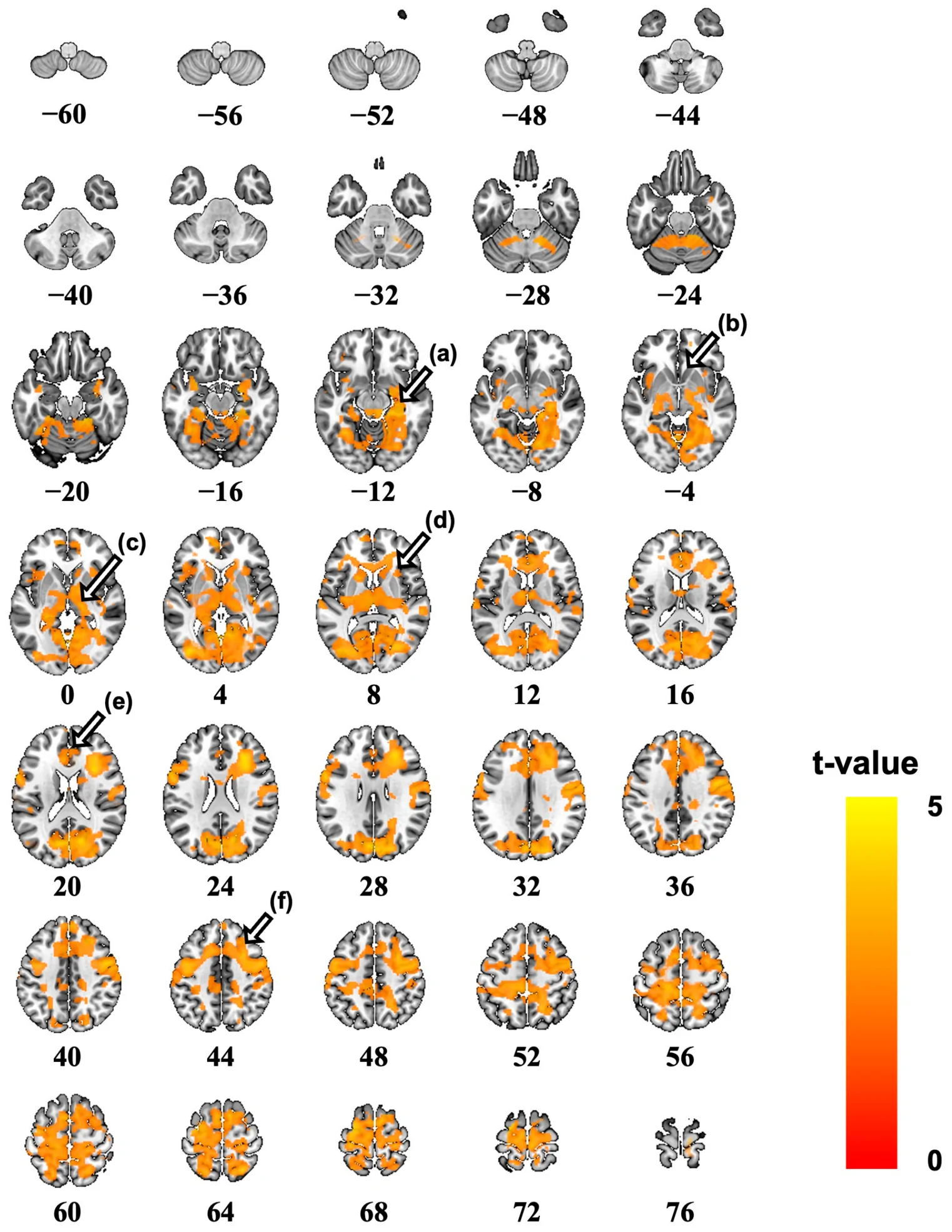
Brain activation maps comparing responses to scented insect versus control scent. Brain regions showing significantly greater activation for the scented insect compared to the control scent, based on paired *t*-test analysis (FDR-corrected *p* < 0.05). Arrows indicate representative anatomical regions showing significant activation: (a) hippocampus, (b) caudate, (c) thalamus, (d) insula, (e) anterior cingulate cortex (ACC), and (f) prefrontal cortex (PFC).

Figure 2 presents the direct comparison between the two scent conditions based on paired *t*-test analysis. Compared with the control scent, exposure to the scented insect elicited significantly greater activation in the thalamus, insula, sensory cortex, hippocampus, and frontal cortical regions, indicating enhanced recruitment of sensory, emotional, and cognitive processing networks (FDR-corrected *p* < 0.05).

### 3.3. Associations Between Brain Activation and Psychometric Scores

**Figure 3:**
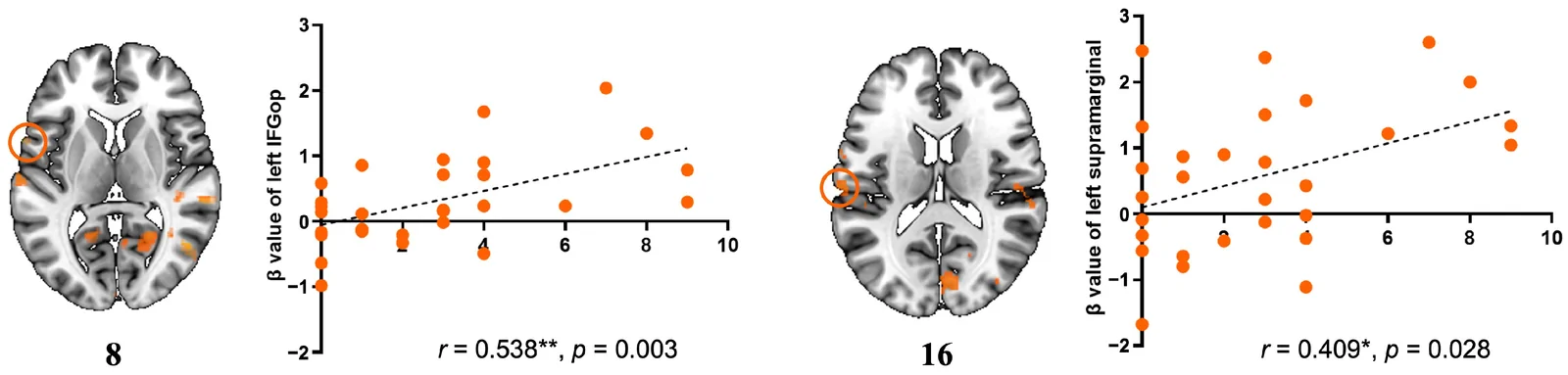
PHQ-9 scores and the left IFGop, and the left supramarginal gyrus. Orange circles indicate the target brain regions, and the dashed line represents the linear regression line. * *p* < 0.05, ** *p* < 0.01.

**Figure 4:**
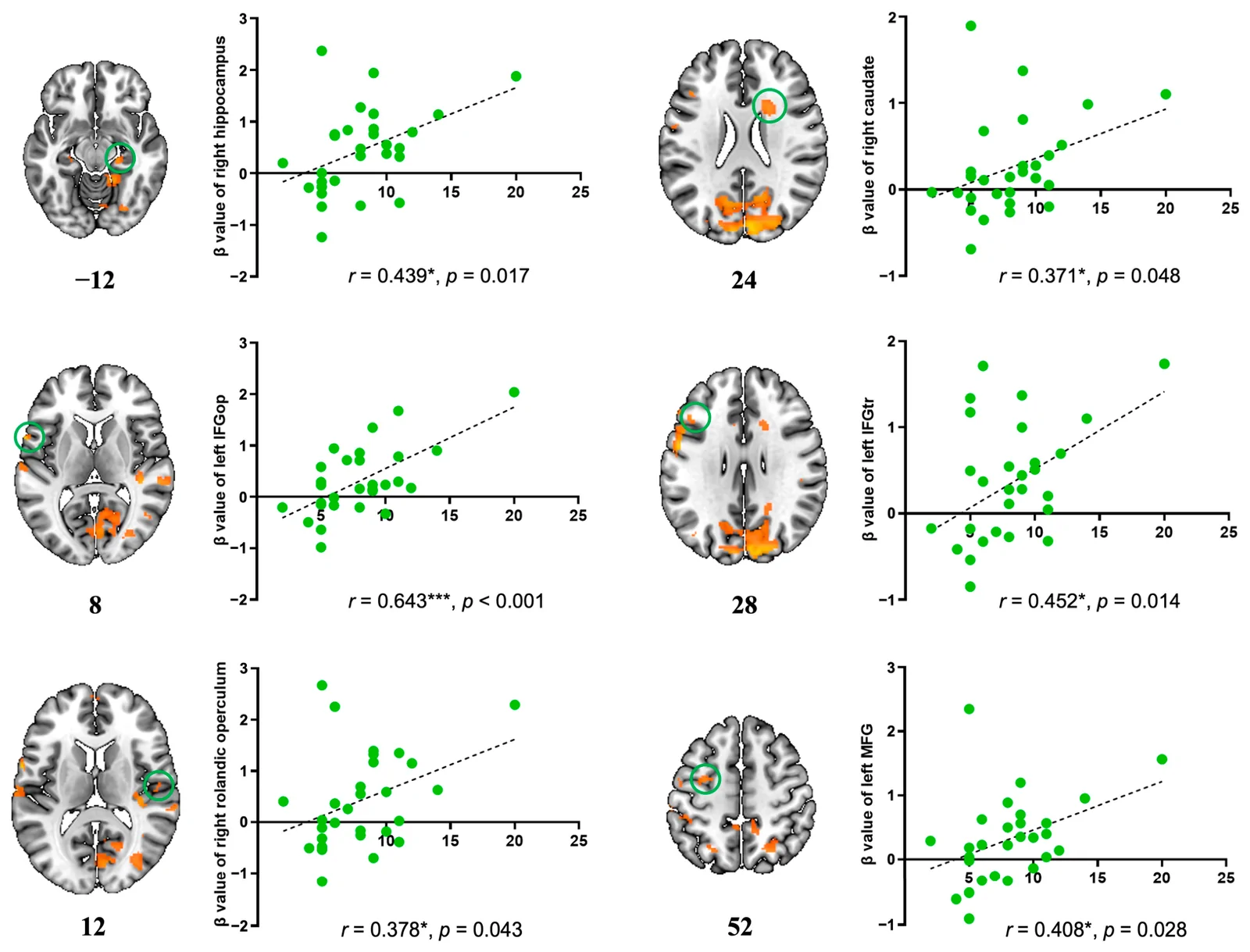
BEPSI scores and the right hippocampus, right Rolandic operculum, left and right IFGtr, right caudate, and left MFG. Green circles indicate the target brain regions, and the dashed line represents the linear regression line. * *p* < 0.05, *** *p* < 0.001.

**Figure 5:**
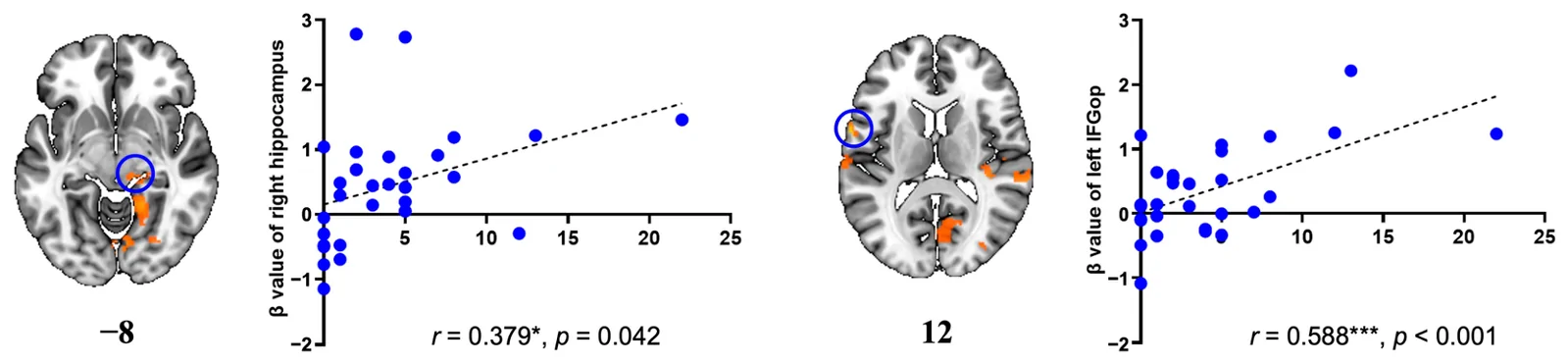
BAI scores and the right hippocampus and left IFGop. Blue circles indicate the target brain regions, and the dashed line represents the linear regression line. * *p* < 0.05, *** *p* < 0.001.

**Figure 6:**
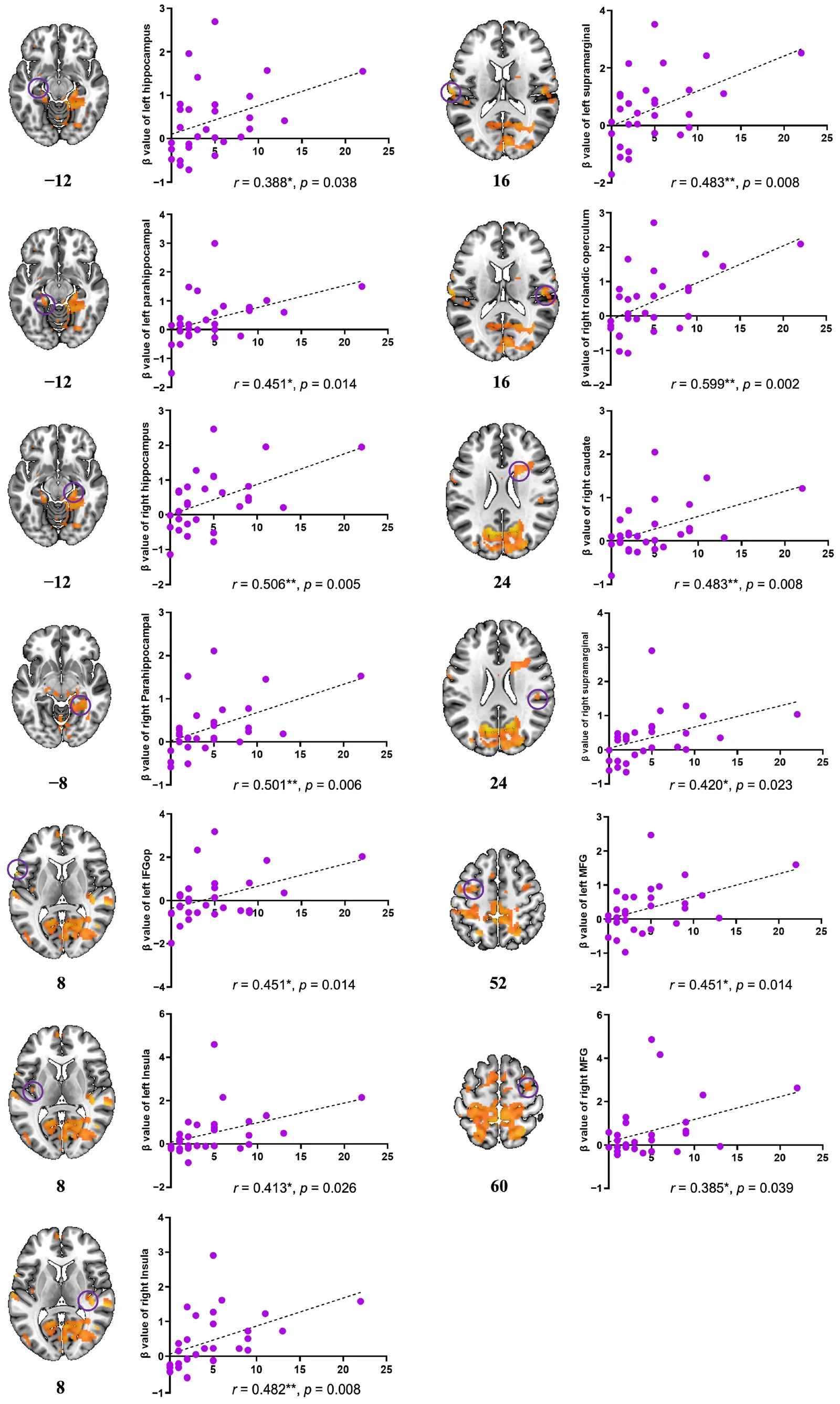
ISI scores and the right caudate, left and right supramarginal gyri, left and right MFG, left and right hippocampi, left and right parahippocampal gyri, left IFGop, left and right insulae, and the Rolandic operculum. Purple circles indicate the target brain regions, and the dashed line represents the linear regression line. * *p* < 0.05, ** *p* < 0.01.

**Figure 7:**
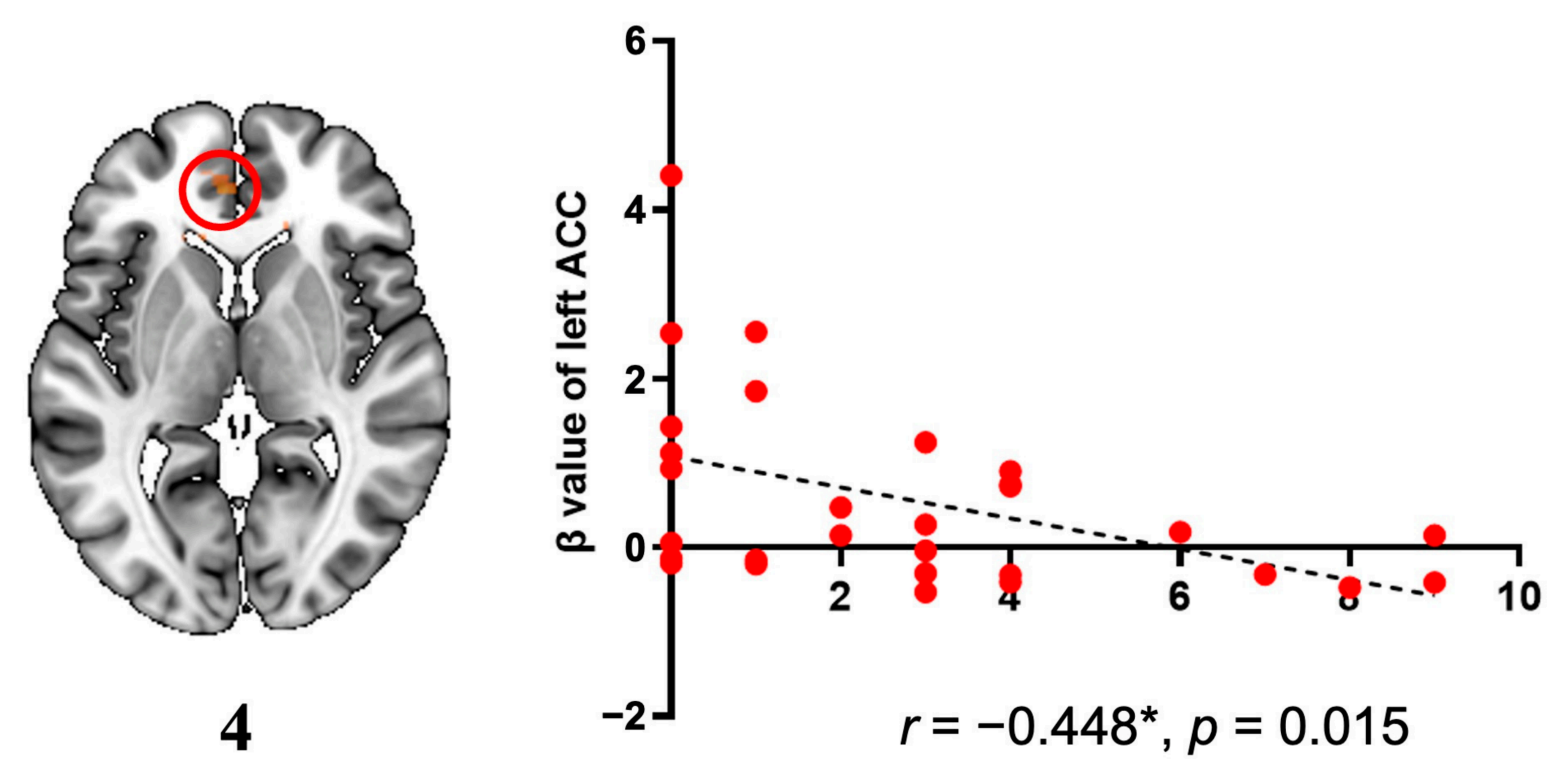
Beta values of brain regions showing significant negative correlations with psychometric scores. A significant negative correlation was observed only between the left ACC and PHQ-9 scores. No other psychometric measures showed significant negative correlations. Red circles indicate the target brain regions, and the dashed line represents the linear regression line. * *p* < 0.05.

Table 2 presents the brain activation maps and corresponding regions identified through multiple regression analysis conducted within the statistical mask defined by the paired *t*-test results (FDR-corrected *p* < 0.05). This analysis assessed brain regions that exhibited significant associations with higher psychometric scores under the scented insect than those under the control scent contrast (*p* < 0.05). Beta values were extracted from the peak coordinates reported in Table 2 for subsequent correlation analyses. Significant moderate positive correlations were observed for all four psychometric assessments: PHQ-9, BEPSI, BAI, and ISI. In contrast, a significant negative correlation was observed only with PHQ-9 scores (Figure 3, Figure 4, Figure 5, Figure 6 and Figure 7, Table 3). Specifically, PHQ-9 scores were positively correlated with the left inferior frontal gyrus (IFG), opercular part (IFGop), and the left supramarginal gyrus (Figure 3). BEPSI scores showed positive associations with the right hippocampus, right Rolandic operculum, bilateral IFG (triangular part, IFGtr), right caudate, and left middle frontal gyrus (MFG) (Figure 4). BAI scores were positively associated with the right hippocampus and left IFGop (Figure 5). ISI scores were positively correlated with the right caudate, bilateral supramarginal gyri, bilateral MFG, bilateral hippocampi, bilateral parahippocampal gyri, left IFGop, bilateral insulae, and the Rolandic operculum (Figure 6). Additionally, the significant negative correlation between Left ACC activation and PHQ-9 scores is presented in Figure 7.

## 4. Discussion

### 4.1. Emerging Role of Animal-Derived Olfaction in Human Neuroscience

To our knowledge, this is the first controlled, crossover-design fMRI study to examine neurological responses to insect-derived olfactory stimulation in humans. Prior studies have suggested that pet insect care can improve cognitive function in elderly individuals [6]. In this randomized controlled trial, community-dwelling seniors who cared for crickets for over 8 weeks exhibited marked improvements in mood and cognitive performance, without notable changes in anxiety, sleep quality, or fatigue. In another fMRI study, elderly women who raised crickets for 8 weeks showed improved executive function accompanied by increased activation in the right dorsolateral prefrontal and parietal cortices [7]. These findings suggest that pet insect care might serve as a form of noncontact AAI to support cognitive health in aging populations. Importantly, the beneficial effects observed in previous insect-rearing studies may reflect multiple factors, including cognitive engagement, routine caregiving activities, emotional attachment, and environmental enrichment, rather than olfactory stimulation alone. Therefore, these studies cannot be considered direct evidence supporting a causal role of insect-derived scents or pheromones in cognitive improvement. To date, no study to date has directly assessed the neurofunctional effects of insect-derived olfactory stimulation in humans using neuroimaging tools such as the electroencephalogram or fMRI. Previous studies on insect-related olfaction have largely focused on comparative neurobiology, investigating the structure and function of insect olfactory systems, or have examined behavioral and psychological responses to insect-related stimuli. Even studies on insect pheromones and their direct effects on human brain function are virtually nonexistent [30,31]. Existing reports have largely addressed behavioral outcomes, such as aversion, discomfort, or allergic responses, rather than underlying neurophysiological mechanisms [32]. In contrast, the therapeutic potential of natural scents derived from plants, particularly aromatic herbs, has been relatively well established. Numerous studies have reported that plant-based olfactory interventions might alleviate insomnia, stress, and anxiety [33]. Still, animal-derived scents remain significantly underexplored in human neuroscience research. In particular, no prior study has investigated human brain responses to insect-derived scents using neuroimaging methods. This gap highlights the novelty and potential significance of the present study.

### 4.2. Neural Activation Patterns Induced by Insect-Derived Scents

Our findings demonstrate that exposure to the naturally emitted scent of a native Korean insect elicited significantly greater activation in multiple brain regions compared to a control scent. These regions were primarily associated with sensory processing, emotion regulation, memory, and cognition, including the thalamus, insula, hippocampus, prefrontal cortex, and supramarginal gyrus. The results indicate a high neurofunctional sensitivity to insect-derived olfactory stimuli within emotion- and cognition-related brain networks, suggesting potential involvement in affective regulation, stress response, and emotional memory processing. Notably, these neural responses were observed even in psychologically healthy individuals, suggesting that insect-derived olfactory stimuli might serve as potential modulators of emotion- and cognition-related brain networks. The consistent activation of sensory and limbic circuits implies a neuromodulatory role of insect scents in regulating affective and cognitive states.

Furthermore, regression analysis results suggest that olfactory stimulation with scented insects might influence brain circuits involved in emotional regulation, memory processing, and sleep-related functions. Across all four psychometric domains—depression, anxiety, stress, and insomnia—higher symptom scores were significantly associated with increased activation in brain regions known to mediate affective and cognitive processing, including the hippocampus, IFG, insula, and parietal areas. These findings suggest that individual psychological characteristics may be associated with variability in neural responses to insect-derived olfactory stimuli.

### 4.3. Symptom-Dependent Neural Sensitivity

Brain regions showing significant positive correlations with depressive symptoms (PHQ-9 scores) included the left opercular part of the IFG (IFGop) and the left supramarginal gyrus (SMG). These regions are implicated in self-referential thinking, emotional interpretation, and linguistic–cognitive emotion processing [34,35,36]. This finding suggests that insect scents might facilitate emotional attribution or memory retrieval in response to olfactory cues, eliciting heightened reactivity in emotion–cognition circuits among individuals with greater depressive tendencies. Notably, IFGop and SMG have been previously linked to emotional hyperreactivity and increased sensitivity to negative emotional processing in patients with major depressive disorder [37]. Individuals with higher BEPSI, BAI, and (ISI) scores exhibited significantly greater activation in brain regions involved in emotion regulation, affective memory, and executive control following exposure to insect-derived olfactory stimuli. Specifically, stress levels were positively associated with activity in the right hippocampus, caudate, IFGtr, and MFG—regions that modulate emotional reactivity and attentional control [38,39,40]. Anxiety scores were correlated with increased activation in the right hippocampus and left IFGop, indicating heightened engagement of memory–emotion circuits and inhibitory control mechanisms in anxiety-prone individuals [41]. Insomnia severity was associated with broader activation across the caudate, hippocampus, parahippocampal gyrus, MFG, insula, supramarginal gyrus, and Rolandic operculum—regions implicated in autonomic regulation, sensory processing, and the sleep–wake cycle [39,42,43,44]. This pattern suggests that individuals with greater sleep disturbance may exhibit amplified neural responses to olfactory input, reflecting heightened sensory sensitivity and dysregulated arousal systems.

A significant negative correlation was observed exclusively for PHQ-9 scores, localized to the left anterior cingulate cortex (ACC). This finding suggests that individuals with higher depressive symptoms might exhibit diminished neural responsivity to insect-derived olfactory stimulation within key emotion regulation networks. This finding aligns with previous studies showing that patients with major depressive disorder often display reduced functional connectivity and activation of the ACC, particularly during negative emotion processing [45]. Thus, the reduced ACC activation in our study potentially reflects blunted affective salience attribution or impaired emotional appraisal in individuals with higher depressive tendencies.

Taken together, these results indicate that insect scent stimulation engages emotion-related neural circuits in a symptom-dependent manner, with the ACC potentially serving as a neural substrate reflecting depressive vulnerability. This finding supports the broader implication that insect-derived olfactory stimuli could potentially be used as noninvasive probes of affective circuit sensitivity in subclinical or clinical depressive states.

### 4.4. Limitations

While this study provides the first neuroimaging evidence that olfactory stimulation with the naturally emitted scent of scented insects robustly activates brain regions involved in sensory integration, emotional regulation, memory, and cognition, several limitations should be considered when interpreting the findings of this study.

First, the sample consisted of a relatively small group of psychologically healthy young Korean adults with a shared ethnic and cultural background. This limited diversity in age, clinical status, and cultural background restricts the external validity (generalizability) of the findings to broader populations, including individuals with diverse psychiatric conditions or from different ethnic and cultural contexts. Future studies should recruit larger, more diverse cohorts to validate these neural responses.

Second, ethyl alcohol was used as the control odor. Although commonly considered neutral, it can evoke mild aversive reactions, suggesting that observed activation patterns might reflect relative rather than absolute effects. Future studies should use more rigorously neutral olfactory controls (e.g., odorless air or deodorized carrier gases) and include plant-based aromas for comparison.

Third, this was a cross-sectional single-session fMRI study, precluding causal interpretation. It remains unclear whether repeated exposure to insect-derived olfactory stimuli produces sustained neurocognitive or emotional benefits. Furthermore, although the observed neural responses correlated with psychological vulnerability markers, these findings alone cannot be used to predict disease progression or symptom onset. Functional brain activation reflects responsiveness to sensory stimuli but does not necessarily indicate a pathological state. In addition, because the study intentionally included only psychologically healthy participants with psychometric scores largely confined to subclinical ranges, the observed brain-behavior correlations should be interpreted within the context of restricted score variability, which may have limited the magnitude of the observed correlation coefficients.

Finally, as this study represents an exploratory first-in-human neuroimaging investigation of insect-derived olfactory stimulation, the findings should be considered preliminary and hypothesis-generating rather than confirmatory. Future studies involving larger independent cohorts, longitudinal designs, repeated exposure paradigms, and additional experimental approaches will be necessary to establish the robustness and generalizability of these observations.

### 4.5. Strengths of the Study

This study has several strengths that enhance its scientific and clinical relevance. First, to our knowledge, it is the first human neuroimaging study to examine brain responses to naturally emitted insect-derived scents using fMRI, investigating the neurofunctional effects of animal-derived scents. Second, by integrating four validated psychometric scales (PHQ-9, BEPSI, BAI, and ISI) and applying multiple regression analyses, the study provided a multidimensional assessment of psychological vulnerability and demonstrated that higher depression, anxiety, stress, or insomnia scores were associated with greater activation in emotion- and cognition-related regions (e.g., hippocampus, IFG, insula). The olfactory stimulus also engaged integrative circuits for emotion regulation, memory, and cognitive control (e.g., thalamus, prefrontal cortex, supramarginal gyrus), indicating that insect-derived scents can activate higher-order affective–cognitive networks beyond primary olfactory processing. These symptom-specific activation patterns highlight the potential of insect-derived scents as natural, noninvasive neuromodulatory stimuli for individuals with emotional vulnerability or sleep disturbances, informing future interventions and translational applications in affective neuroscience and mental health. Finally, the study employed rigorous statistical methods, including FDR-corrected paired *t*-tests and voxelwise multiple regression analyses within predefined activation masks, ensuring anatomical specificity and statistical reliability.

## 5. Conclusions

This study provides the first neuroimaging evidence that olfactory stimulation with a scented insect robustly engages a broad network of brain regions involved in sensory processing, emotional regulation, memory, and cognition. These neural responses were consistently observed even in psychologically healthy individuals, suggesting that insect-derived odors might exert intrinsic neuromodulatory effects, independent of subjective hedonic appraisal. Furthermore, multiple regression analyses showed that brain responses to the insect scent vary with individual psychological vulnerability, with higher scores on measures of depression, anxiety, stress, and insomnia associated with greater activation in key emotion–cognition circuits, including the hippocampus, IFG, and insula. These findings suggest that insect-derived olfactory stimuli may provide a useful framework for exploring individual variability in neural responses to affective and cognitive processing.

The symptom-specific activation patterns underscore the potential of insect-derived olfactory cues as novel, noninvasive neuromodulatory tools for emotional and cognitive dysregulation. Future studies should examine the clinical utility of insect scents in populations with mood and sleep disorders and directly compare their effects with established plant-based aromas, such as lavender and herbal essential oils. To establish the therapeutic efficacy of insect scents, studies incorporating objective physiological and behavioral markers are needed.

## Figures and Tables

**Table 1:** Participant characteristics and scent preference ratings.

Characteristic		Mean
Total		29
Gender		
	Male	8 (27.6%)
	Female	21 (72.4%)
Age		28.2 ± 6.1
PHQ-9		2.8 ± 2.8
BEPSI		1.6 ± 0.7
BAI		3.9 ± 4.1
ISI		4.5 ± 4.0
Sleep duration		7.2 ± 1.1
Preference for scent *		
	Scented insects	4.0 ± 0.8
	Control	2.8 ± 0.9

* 5-point Likert scale: 1 = strongly dislike, 5 = strongly like; a paired *t*-test showed a significant difference in preference between the two scents (*p* < 0.001).

**Table 2:** Brain regions associated with psychometric scores. Results from multiple regression analysis within the paired *t*-test mask (scented insect > control; FDR-corrected *p* < 0.05).

Brain Region	Side	Cluster Size	MNI Coordinates			Peak T
			*x*	*y*	*z*	
Covariate: PHQ-9						
Anterior cingulate cortex	L	14	−4	44	4	2.83
Inferior frontal gyrus, opercular part	L	16	−60	6	8	3.45
Supramarginal	L	15	−66	−22	16	2.37
Covariate: BEPSI						
Caudate	R	17	22	8	22	2.34
Hippocampus	R	28	22	−26	−12	2.86
Inferior frontal gyrus, opercular part	L	20	−60	6	8	4.27
Inferior frontal gyrus, triangular part	L	23	−42	24	26	2.78
Middle frontal gyrus	L	14	−30	−6	50	2.50
Rolandic operculum	R	19	50	−16	14	2.46
Covariate: BAI						
Hippocampus	R	26	16	−28	−8	2.26
Inferior frontal gyrus, opercular part	L	21	−62	6	10	3.81
Covariate: ISI						
Caudate	R	51	22	6	22	3.12
Hippocampus	L	18	−24	−26	−12	2.22
	R	47	22	−24	−12	3.26
Inferior frontal gyrus, opercular part	L	19	−62	8	8	4.69
Insula	L	21	−40	−14	8	2.44
	R	44	38	−20	8	2.90
Middle frontal gyrus	L	27	−30	−6	52	2.74
	R	103	36	−2	60	2.18
Parahippocampal	L	22	−20	−34	−12	2.71
	R	137	30	−42	−8	3.24
Rolandic operculum	R	84	50	−20	16	3.72
Supramarginal	L	16	−60	−22	16	2.82
	R	103	48	−30	26	2.59

**Table 3:** Brain regions showing significant correlations between extracted beta values from 4-mm-radius spherical ROIs (centered at the peak coordinates identified in the masked multiple-regression analysis; Table 2) and psychometric scores (PHQ-9, BEPSI, BAI, ISI). Reported values include MNI coordinates, Pearson’s correlation coefficients (*r*), and corresponding *p* values.

Brain Region	Side	MNI Coordinates			*r*	*p*
		*x*	*y*	*z*		
Covariate: PHQ-9						
Anterior cingulate cortex	L	−4	44	4	−0.448	0.015
Inferior frontal gyrus, opercular part	L	−60	6	8	0.538	0.003
Supramarginal	L	−66	−22	16	0.409	0.028
Covariate: BEPSI						
Caudate	R	22	8	22	0.371	0.048
Hippocampus	R	22	−26	−12	0.439	0.017
Inferior frontal gyrus, opercular part	L	−60	6	8	0.643	<0.001
Inferior frontal gyrus, triangular part	L	−42	24	26	0.452	0.014
Middle frontal gyrus	L	−30	−6	50	0.408	0.028
Rolandic operculum	R	50	−16	14	0.378	0.043
Covariate: BAI						
Hippocampus	R	16	−28	−8	0.379	0.042
Inferior frontal gyrus, opercular part	L	−62	6	10	0.588	<0.001
Covariate: ISI						
Caudate	R	22	6	22	0.483	0.008
Hippocampus	L	−24	−26	−12	0.388	0.038
	R	22	−24	−12	0.506	0.005
Inferior frontal gyrus, opercular part	L	−62	8	8	0.451	0.014
Insula	L	−40	−14	8	0.413	0.026
	R	38	−20	8	0.482	0.008
Middle frontal gyrus	L	−30	−6	52	0.451	0.014
	R	36	−2	60	0.385	0.039
Parahippocampal	L	−20	−34	−12	0.451	0.014
	R	30	−42	−8	0.501	0.006
Rolandic operculum	R	50	−20	16	0.599	0.002
Supramarginal	L	−60	−22	16	0.483	0.008
	R	48	−30	26	0.420	0.023

## Data Availability

The data supporting the findings of this study are available from the corresponding author upon reasonable request. The data are not publicly available due to privacy and ethical restrictions related to human participant data.

## Supplementary Materials

The following supporting information can be downloaded at: https://www.mdpi.com/article/10.3390/insects17080761/s1, Figure S1: Whole brain activation map during exposure to the scented insect. Figure S2: Whole brain activation map during exposure to the scented control. Table S1: Peak coordinates of brain regions activated during exposure to the scented insect. Table S2: Peak coordinates of brain regions activated during exposure to the scented control.

## Abbreviations

The following abbreviations are used in this manuscript:

AAI	Animal-Assisted Intervention
ACC	Anterior Cingulate Cortex
BAI	Beck Anxiety Inventory
BEPSI	Brief Encounter Psychosocial Instrument
BOLD	Blood Oxygenation Level–Dependent
CAT12	Computational Anatomy Toolbox
EPI	Echo Planar Imaging
FA	Flip Angle
FDR	False Discovery Rate
FOV	Field of View
fMRI	Functional Magnetic Resonance Imaging
FWHM	Full Width at Half Maximum
GLM	General Linear Model
IFG	Inferior Frontal Gyrus
IFGop	Inferior Frontal Gyrus, Opercular Part
IFGtr	Inferior Frontal Gyrus, Triangular Part
ISI	Insomnia Severity Index
MFG	Middle Frontal Gyrus
MNI	Montreal Neurological Institute
MRI	Magnetic Resonance Imaging
PHQ-9	Patient Health Questionnaire-9
ROI	Region of Interest
SMG	Supramarginal Gyrus
SPM12	Statistical Parametric Mapping (version 12)
TE	Echo Time
