# Supplementary material for: Neurological Responses to Scented Insects via Olfactory Stimulation: A Controlled fMRI Study
Source: Insects. 2026 Jul 24;17(8):761. doi: 10.3390/insects17080761 (PMC13513610; doi:10.3390/insects17080761)
Supplement: Supplementary file 1 [file insects-17-00761-s001.zip › insects-4369516-supplementary.pdf]

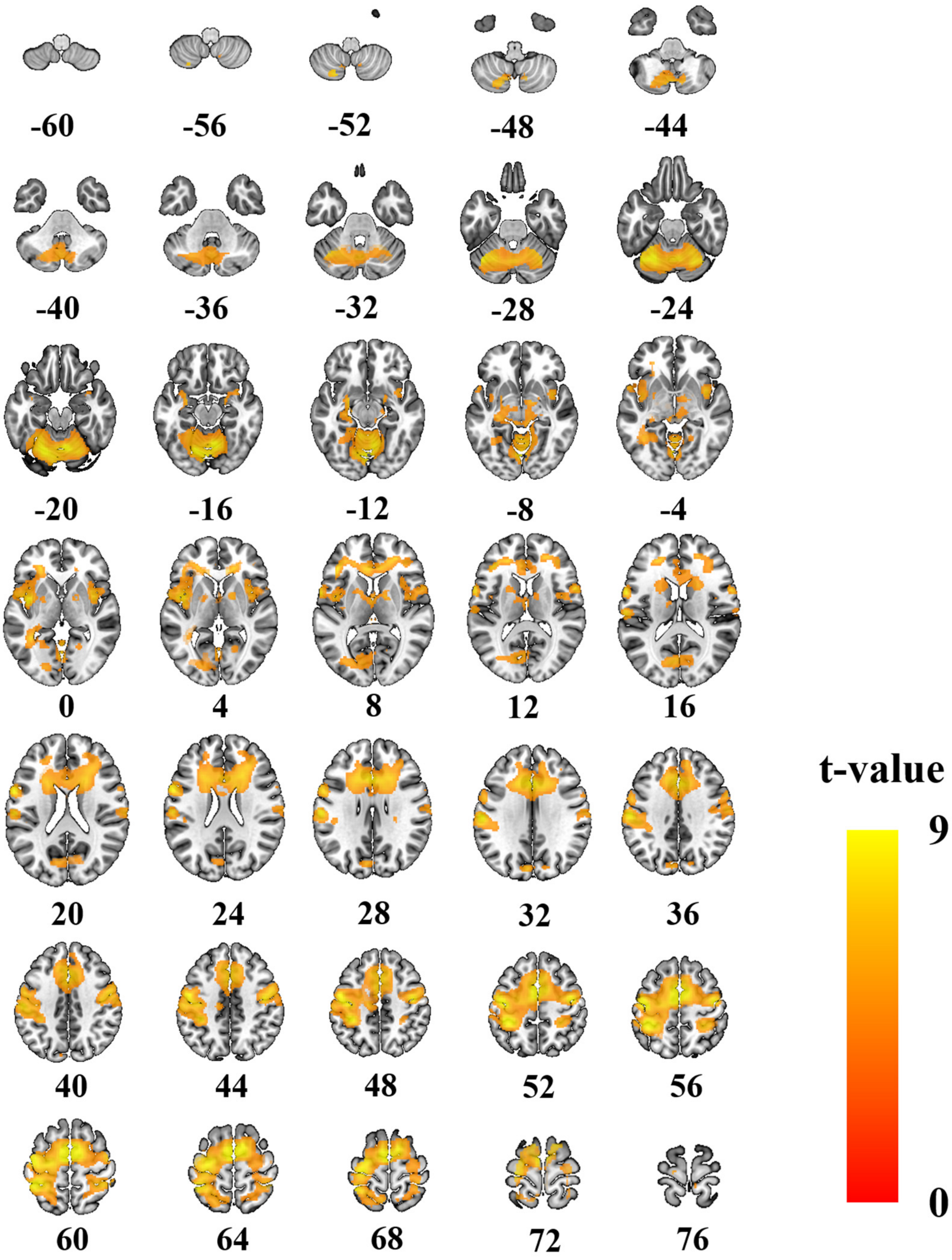

Supplementary Figure S1. Whole brain activation map during exposure to the scented insect. Voxel-wise one-sample *t*-test showing brain regions significantly activated during exposure to the scented insect odor (FDR-corrected $p < 0.05$).

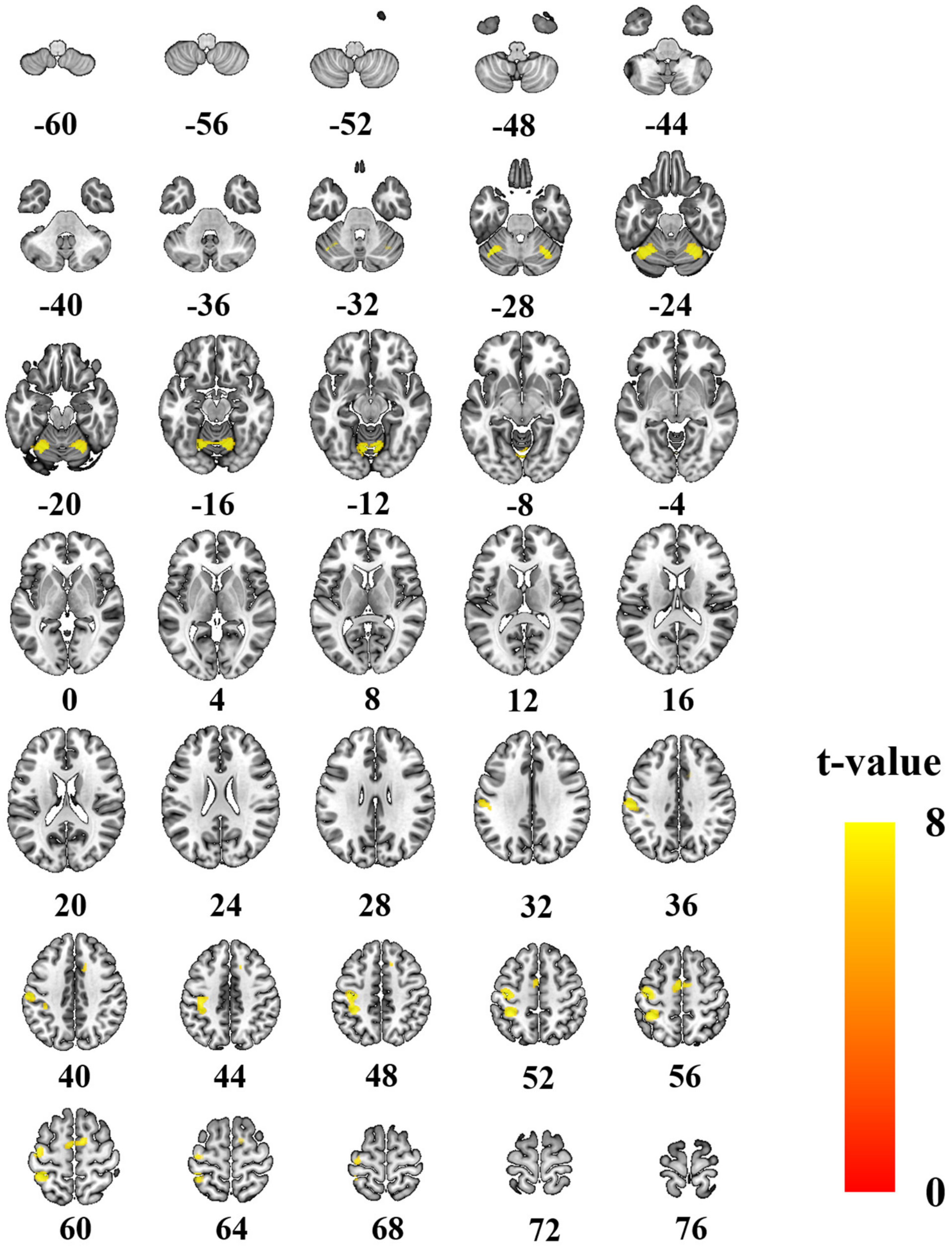

Supplementary Figure S2. Whole brain activation map during exposure to the scented control. Voxel-wise one-sample *t*-test showing brain regions significantly activated during exposure to the scented control.

Supplementary Table S1. Peak coordinates of brain regions activated during exposure to the scented insect. (FDR-corrected $p < 0.05$)

Brain region	Side	Cluster size	MNI-coordinates			Peak T
			x	*y*	*z*	
Amygdala	L	21	-24	-8	-12	4.93
	R	36	22	-2	-16	4.07
Anterior cingulate cortex	L	72	-6	38	6	3.59
	R	36	16	40	8	4.02
Caudate	L	103	20	12	20	4.36
	R	132	-18	8	20	3.82
Cuneus	L	321	-6	-82	32	4.48
	R	54	16	-80	34	3.52
Hippocampus	L	203	-22	-24	-12	4.21
	R	18	18	-24	-10	3.33
Inferior frontal gyrus, opercular part	L	266	-58	8	10	3.62
	R	101	56	10	10	3.97
Inferior frontal gyrus, triangular part	L	302	-40	34	10	5.55
	R	50	42	38	8	3.86
Inferior parietal lobule	R	66	38	-38	52	3.71
	L	898	-38	-38	52	6.65
Insula	R	622	40	4	4	3.88
	L	472	-40	-2	0	5.75
Middle cingulate cortex	R	650	8	16	32	5
	L	698	0	20	32	5.73
Middle frontal cortex	R	164	30	32	22	4.61
	L	438	-26	32	26	3.32
Middle temporal gyrus	L	32	-42	-54	-4	3.78
Parahippocampal	L	25	-24	-30	-12	3.31
Precuneus	L	131	-8	-56	70	4.47
	R	21	8	-58	66	3.25
Rolandic operculum	L	251	-60	2	8	5.18
	R	260	60	6	14	5.21
Superior frontal gyrus	R	924	16	2	58	4.59
	L	899	-22	-6	60	3.99
Superior medial frontal gyrus	L	427	-8	28	34	4.45
	R	228	10	40	34	4.33
Superior parietal lobule	R	622	-20	-62	64	4.02
	L	279	20	-54	66	3.57
Superior temporal gyrus	L	87	-62	-18	10	3.42
Superior temporal pole	L	95	-56	8	-2	4.23
Supramarginal	R	192	66	-18	20	4.27
	L	495	-62	-22	32	5.76

Supplementary Table S2. Peak coordinates of brain regions activated during exposure to the scented control.

Brain region	Side	Cluster size	MNI-coordinates			Peak T
			x	*y*	*z*	
Inferior parietal lobule	L	166	-36	-40	52	5.93
Middle cingulate cortex	R	16	12	12	40	4.43
Superior parietal lobule	L	61	-36	-46	60	5.17
Supermarginal	L	126	-56	-22	36	6.13
